# Red clays indicate sub-aerial exposure of the Rio Grande Rise during the Eocene volcanic episode

**DOI:** 10.1038/s41598-023-46273-y

**Published:** 2023-11-04

**Authors:** Priyeshu Srivastava, Bramley J. Murton, Lucy Gomes Sant’Anna, Fabio Florindo, Muhammad Bin Hassan, Julia Taciro Mandacaru Guerra, Valdecir de Assis Janasi, Luigi Jovane

**Affiliations:** 1 Instituto Oceanográfico, Universidade de São Paulo, Praça do Oceanográfico, 191, São Paulo, 05508-120 Brazil; 2 Indian Institute of Geomagnetism, Navi Mumbai, 410218 India; 3 Istituto Nazionale di Geofisica e Vulcanologia, Via di Vigna Murata 605, 00143 Rome, Italy; 4 National Oceanography Centre, European Way, Southampton, SO14 3ZH UK; 5 Instituto de Energia e Ambiente, Universidade de São Paulo, Av. Prof. Luciano Gualberto, 1289, São Paulo, 05508-010 Brazil; 6 Instituto de Geociências, Universidade de São Paulo, Rua do Lago, 562, São Paulo, 05508-080 Brazil

**Keywords:** Ocean sciences, Solid Earth sciences, Geochemistry, Geology, Mineralogy, Palaeomagnetism

## Abstract

Autonomous underwater vehicle (AUV) mapping of the western Rio Grande Rise (RGR), South Atlantic, and subsequent exploration and photography of horizontal lava flows exposed in near vertical, faulted escarpments, showed occurrences of red clays/weathered volcanic tops trapped between successive alkaline lava flows. These red clays indicate a hiatus in successive volcanic eruptions. Here, we report detailed mineralogical, geochemical, and rock magnetic characteristics of one such distinct red clay dredged from ~ 650 m water depth in the western RGR. The mineral constituents of the red clay are kaolinite, magnetite, oxidized magnetite (/maghemite), hematite, and goethite, with biogenic calcite and halite occupying voids or precipitated on the surface of the red clay. The chemical index of alteration (CIA) has a value of 93, showing that red clay is a product of extreme chemical weathering of the lava flows. The alkaline volcanic rocks recovered from nearby show an age of ~ 44 Ma, indicating an Eocene age for the volcanism. We show that the red clays are a product of sub-aerial chemical weathering of these Eocene volcanic rocks, in a warm-wet climate, before the thermal subsidence of the RGR to its modern-day bathymetric depth.

## Introduction

### Rio Grande Rise evolution

**Figure 1:**
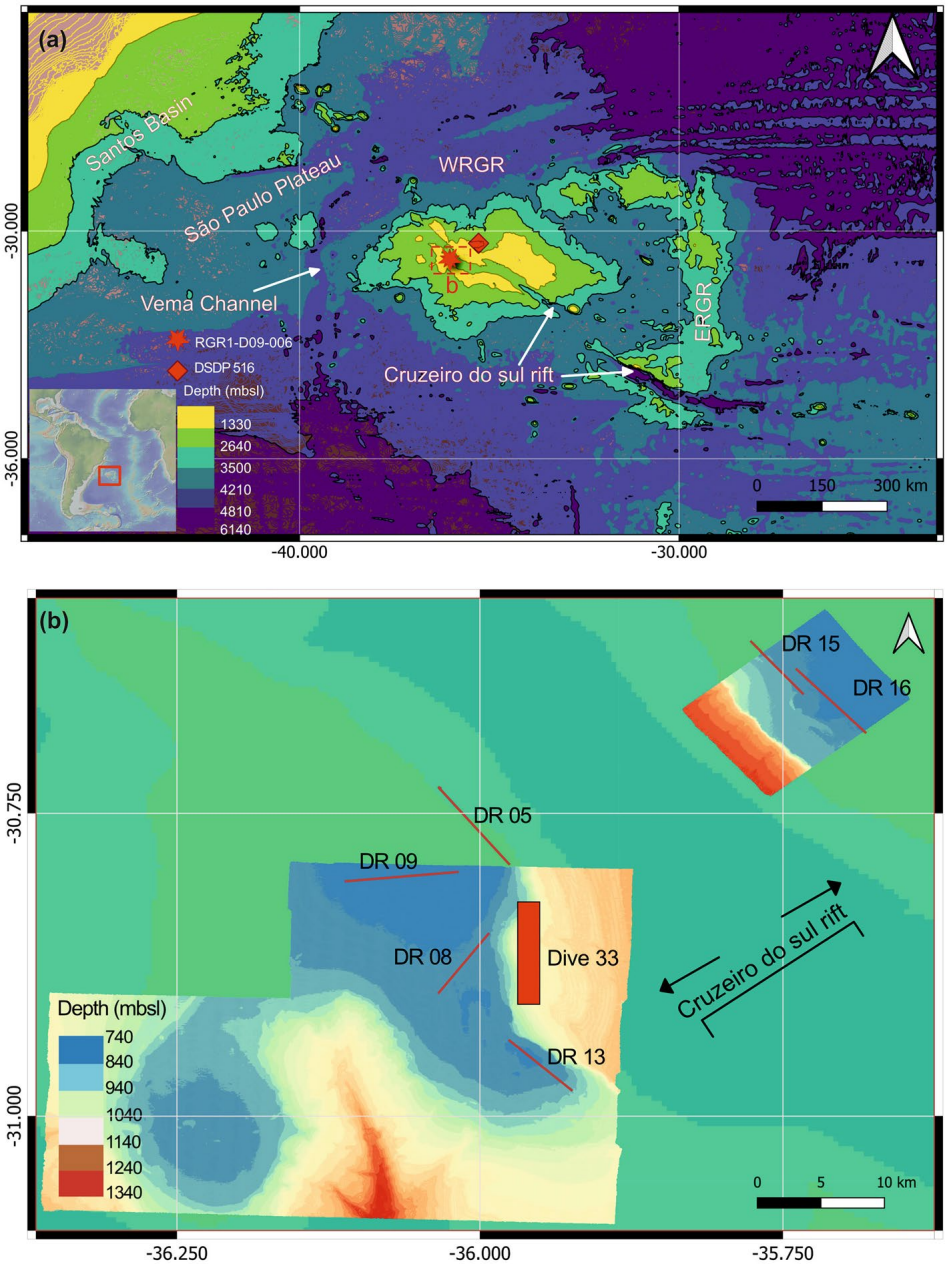
(**a**) The bathymetric map of the Rio Grande Rise (RGR), central South Atlantic showing dredging location of the red clay (star) along with DSDP Site 516 (rectangle); (**b**) the high resolution bathymetry of the study area in the WRGR with locations of different dredges of red clay and alkaline volcanic rocks explored on board Alpha Crucis, and thick red colored line shows the area mapped (Dive 33) using AUV-HyBIS submarine camera on board RRS Discovery.

The Rio Grande Rise (RGR) and Walvis Ridge (WR) are the two prominent large igneous provinces of the South Atlantic. They formed on the South American and African tectonic plates, respectively, by the Tristan-Gough mantle plume situated beneath or in close proximity to the Mid-Atlantic Ridge (MAR) during the Late Cretaceous^1–4^. The RGR is located ~ 1200 km off the SE Brazilian coast and about 2000 km west of the MAR and is divided into western RGR (WRGR) and eastern RGR (ERGR) (Fig. 1a)^5^. Structurally, the RGR is a large intraplate aseismic oceanic plateau that rises from the water depths of about ~ 5000 m to bathymetries less than 600 m in some parts of the WRGR^6^.

The opening of the South Atlantic and the evolution of the RGR are a result of complex tectonic–magmatic processes^7^. Plate reconstruction and tectonic evolution model of the South Atlantic indicate that RGR (basaltic basement) began forming during the end of magnetic Chron C34n (~ 92 Ma), which was followed by the MAR reorganization and eastward ridge jump between magnetic anomaly C34 (~ 83.6 Ma) and C31 (~ 69.2 Ma)^7^. ^40^Ar–^39^Ar ages of the tholeiitic basalts of the WRGR basement recovered from the Deep-Sea Drilling Project (DSDP) Site 516 range from ~ 87 to 80 Ma^3,8,9^. While, the N–S trending ERGR was formed between ~ 83 and 70 Ma^3,4,7,9^, and has similar geochemical affinity as of tholeiitic lava flows of the WRGR (DSDP Site 516) and WR^10^.

The Late Cretaceous (~ 70 Ma) to middle Eocene (~ 50 Ma) extensional tectonics in the RGR led to the development of deep-seated NW–SE trending Cruzeiro do Sul Rift (CdSR) and graben-like structures in the WRGR^11^. The WRGR records a second magmatic (alkaline) event during the Eocene (~ 46–44 Ma)^9,12^, ~ 25–30 Ma after the main shield-stage tholeiitic volcanism at the MAR. Hoyer et al.^10^ suggested that the alkaline volcanic rocks from the second magmatic event have compositions similar to Ocean Island Basalt (OIB), and similar to the Jean Charcot Seamount Chain (JCSC) lavas, a chain of volcanic seamounts in the northwestern extension of CdSR, and linked the second RGR magmatism phase to the Eocene rifting related volcanism. The Eocene magmatic events led to the broad rise of the WRGR^5,11^. The upper Eocene to Oligocene (~ 35 to 25 Ma) period in the RGR evolution phase records tectonic-volcanic quiescence, thermal subsidence, sub-aerial erosion of the WRGR, and extensive sedimentation^5,11^. Subsequently, deep to shallow sub-vertical normal faults had occurred in the RGR plateau region during the Miocene and Pleistocene which led to the formation of > 500 m wide depressions at the RGR sea floor^11^.

## Sub-aerial exposure of the Rio Grande Rise

The RGR has been suggested to have sub-aerially exposed during the volcanism, like a large island, subsequently followed by erosion, thermal subsidence, and pelagic sedimentation^13^. Thiede^14^, based on backtracking paleo-depth information assuming the RGR has subsided at the normal rate as of other oceanic basements, suggested that the RGR towered above the sea-level by over 2 km during the Late Cretaceous volcanism near the MAR. Further studies accounting for the Eocene volcanism, as well as based on paleontological data from the DSDP sites 516 and 357, reported that the RGR was formed near sea-level (shallow depth pelagic sedimentation < 20 m water depth) during the Late Cretaceous volcanism at MAR^5^ or only had sub-aerially exposure at ~ 180 m above sea-level^15^. The seismic and pelagic sedimentation data from the WRGR suggested the guyots and seamounts formed during the Eocene alkaline volcanism were also exposed sub-aerially^5,6,11,15,16^. However, there remains significant uncertainty about the position of the WRGR during the Eocene and whether it was sub-aerial or not.

**Figure 2:**
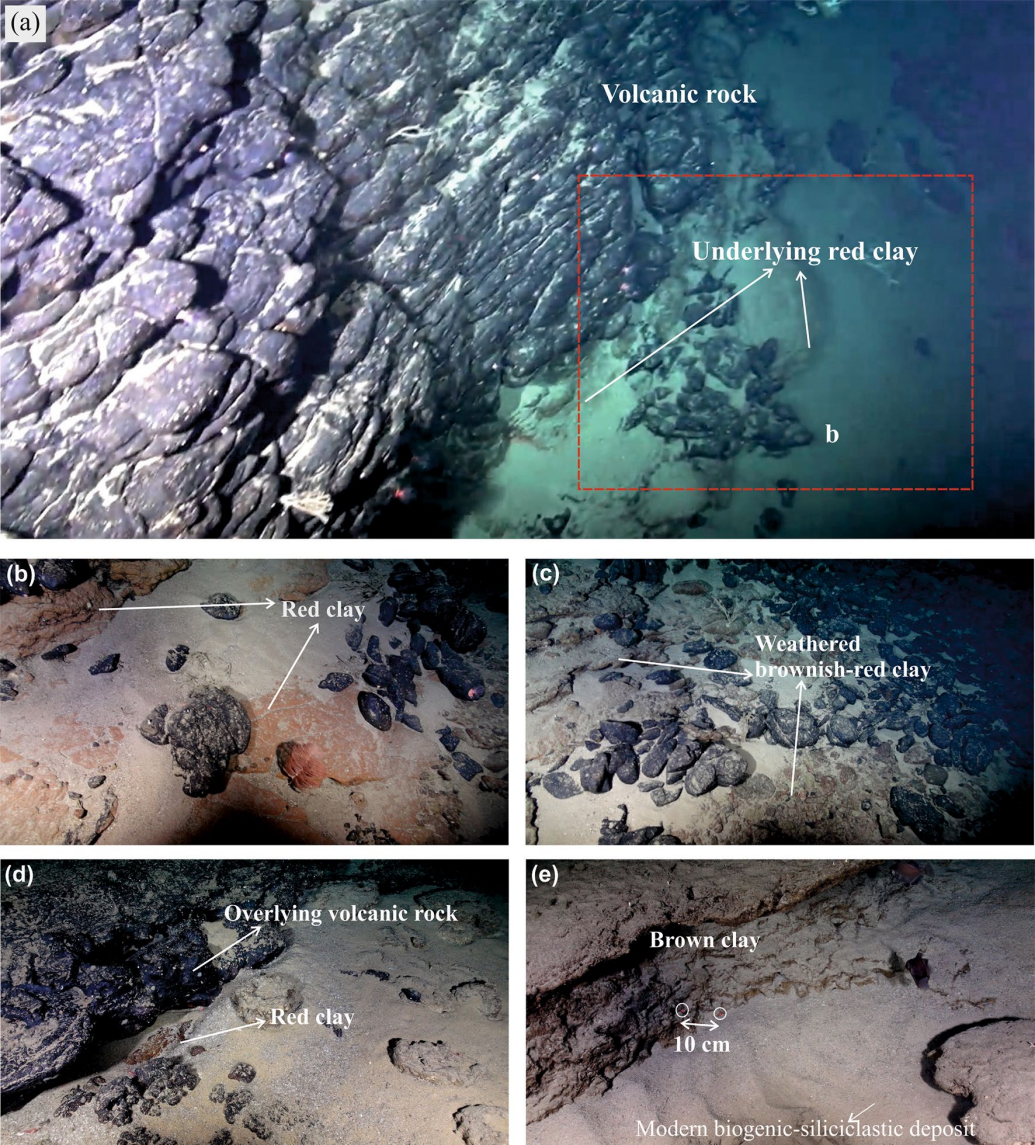
High-definition images captured by the ROV HyBIS on the dive number 33 showing different red and brown colored clays between dark-colored lava flow exposed on the southern scarp of the Cruzeiro do Sul Rift. (**a**) Thick vertical volcanic rock escarpment overlain on the red colored clay; (**b**) enhanced image of the red clay shown in the (**a**); (**c**) thick weathered brownish-red clay; (**d**) very thin exposed red clay overlain by massive volcanic rock; (**e**) exposed brown clay with two laser red dots. The laser dots are 10 cm apart indicating the thickness of red and brown clays are > 10 cm at several places. The greyish silt-sandy deposits seen at all sites are modern siliciclastic-biogenic deposits.

Two multi-disciplinary scientific expeditions in January–February 2018 and in October–November 2018 were conducted in the WRGR on board the Brazilian R/V Alpha Crucis (Instituto Oceanográfico, Universidade de São Paulo), and Royal Research Ship (RRS) Discovery, United Kingdom (UK) (in partnership with Instituto Oceanográfico, Universidade de São Paulo), respectively (Fig. 1). Bathymetry and dredging along the WRGR were carried out on board the R/V Alpha Crucis, and several volcanic rocks including red clay were recovered (Table S1)^17^. Detailed bathymetry and sidescan sonar surveys of the sea floor were made using autonomous underwater vehicle (AUV) Autosub6000, and high-resolution video surveys were made using the remotely operated vehicle HyBIS from the mother ship, RRS Discovery. The detailed mapping of volcanic escarpments of the WRGR showed occurrences of red clays/weathered volcanic tops trapped between successive lava flows (Fig. 2). We studied mineralogical composition, geochemistry, and rock magnetic properties of the red clay to understand its origin and significance in the evolution of RGR.

## Results

### Mineralogy

**Figure 3:**
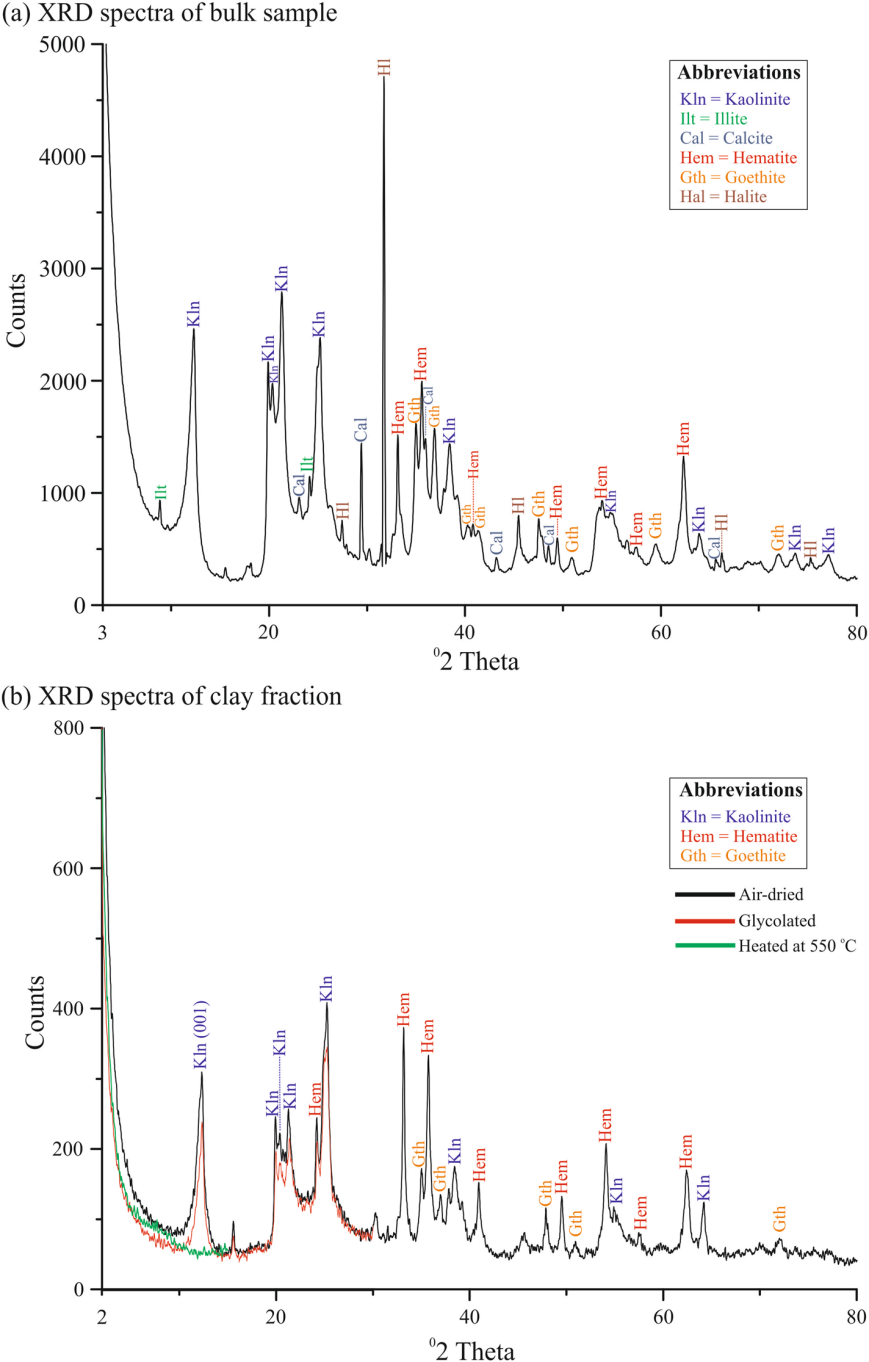
XRD spectra of bulk red clay sample (**a**), and clay separated fraction with different treatments (**b**).

The XRD spectra of the bulk red clay sample is presented in Fig. 3a (Table S2 summarizes the d-spaces of identified minerals) and shows it is composed of kaolinite, illite, halite, calcite, hematite, and goethite (Fig. 3a). Semi-quantitative XRD analysis on oriented clay mounts shows kaolinite as dominant clay mineral (Fig. 3b). No hydrous-group of clays (e.g., smectite) were found upon glycolation. The XRD spectra of sample calcined at ~ 500 °C showed loss of 001 peak confirming kaolinite (Fig. 3b).

**Figure 4:**
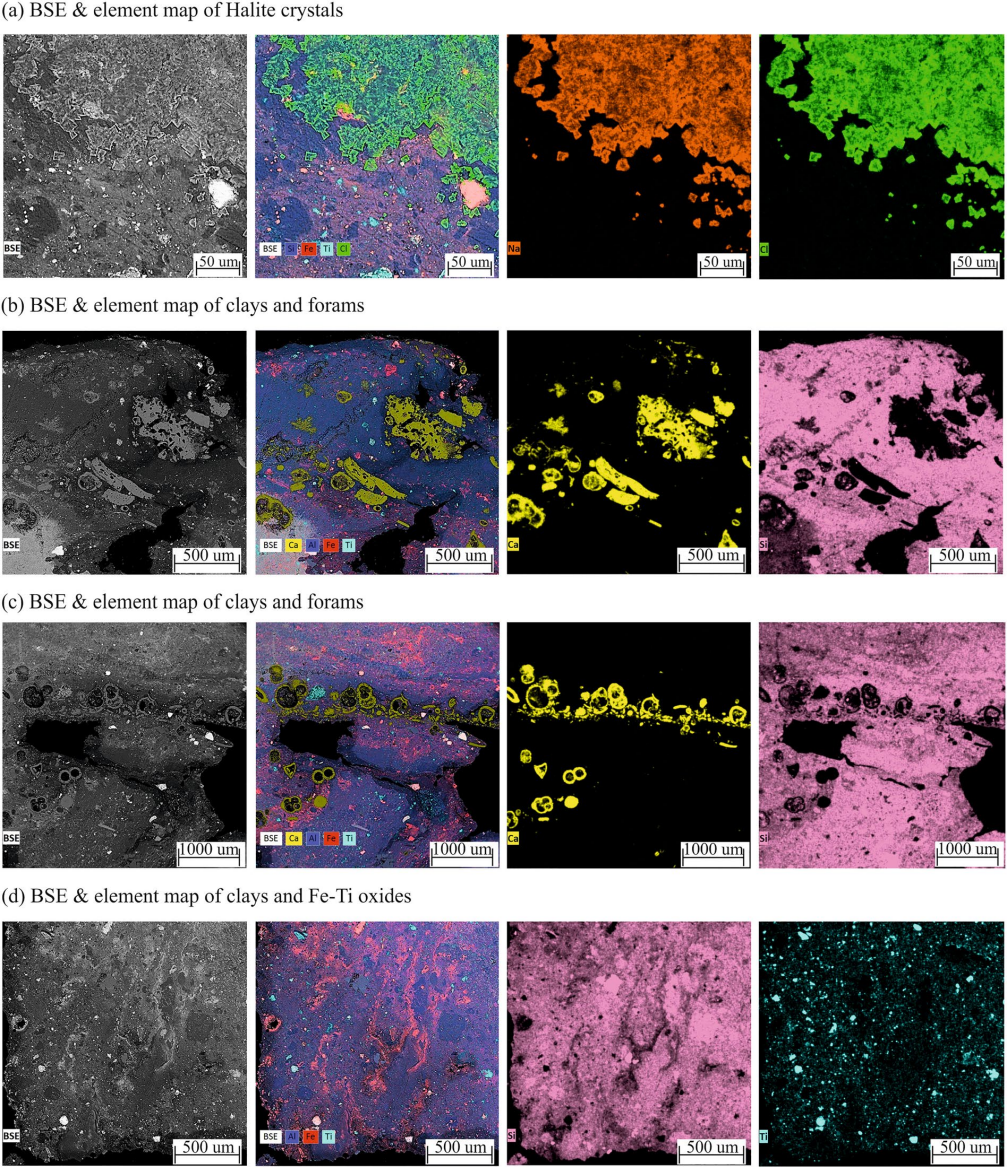
Back-scattering images (BSE) and elemental maps of different compositional components and matrix of the red clay.

SEM and element maps of the red clay are shown in Fig. 4, where salt crystals (halite) and abundant foraminifera are seen (Fig. 4a–c). However, these do not appear to be syngenetic with the clay formation as they are either found above the clay horizon (Fig. 4a) or occupying interstitial spaces and voids (Fig. 4b, c). Lithogenic Ti–rich oxides and secondary iron oxide precipitates were also seen (Fig. 4d). The EDX spectra for groundmass and lithic fragments is provided in the Figs. S1–S3. The groundmass is dominantly composed of Al–Si clay (kaolinite) and lithic fragments are Fe–Ti oxides (e.g., titanomagnetite).

### Geochemistry

The major element data of the bulk red clay sample showed the following order of decreasing concentration (wt%) SiO_2_ (30.7%) > Al_2_O_3_ (24.8%) > Fe_2_O_3_ (15.6%) > TiO_2_ (3.42%) > CaO (1.86%) > MgO (0.92%) > Na_2_O (0.84%) > P_2_O_5_ (0.43%) > K_2_O (0.29%) > MnO (0.1%). The high concentration of Al, Si, Fe and Ti elements indicates that the red clay sample is enriched in Al-Si phyllosilicates, and Fe–Ti oxides.

The calcium carbonate concentration was calculated using the following equation:

1 $${\text{CaCO}}_{{3}} = { 1}.{78 } \times {\text{ CaO}} - {\text{CaO}}*$$

where CaO refers to the bulk sample, and CaO* to the silicate fraction of sample (i.e., CaO measured in samples after carbonate removal from 1N HCl treatment).

The CaCO_3_ weight percentage was ~ 3.13, indicating low carbonate component in the red clay. The LOI of bulk red clay sample was ~ 19.9%.

The chemical index of alteration (CIA) was calculated using the following equation given by Nesbitt and Young^18^:

2 $${\text{CIA }} = { }100 \times \left( {\frac{{{\text{Al}}_{2} {\text{O}}_{3} }}{{{\text{Al}}_{2} {\text{O}}_{3} + {\text{CaO}}^{*} + {\text{K}}_{2} {\text{O}} + {\text{Na}}_{2} {\text{O}}}}} \right)$$

Here, major oxides concentrations are first converted into moles. CaO* represents concentration in silicate fraction and is corrected for carbonate and apatite.

As chemical weathering increases, more labile cations (e.g., Ca^+2^, K^+^ and Na^+^) are leached from primary minerals (e.g., feldspar) of parent rocks and stable residual elements (e.g., Al^+3^) are retained forming secondary clays^18,19^. The CIA value of the red clay is 93, indicating extreme chemical weathering of red clay.

**Figure 5:**
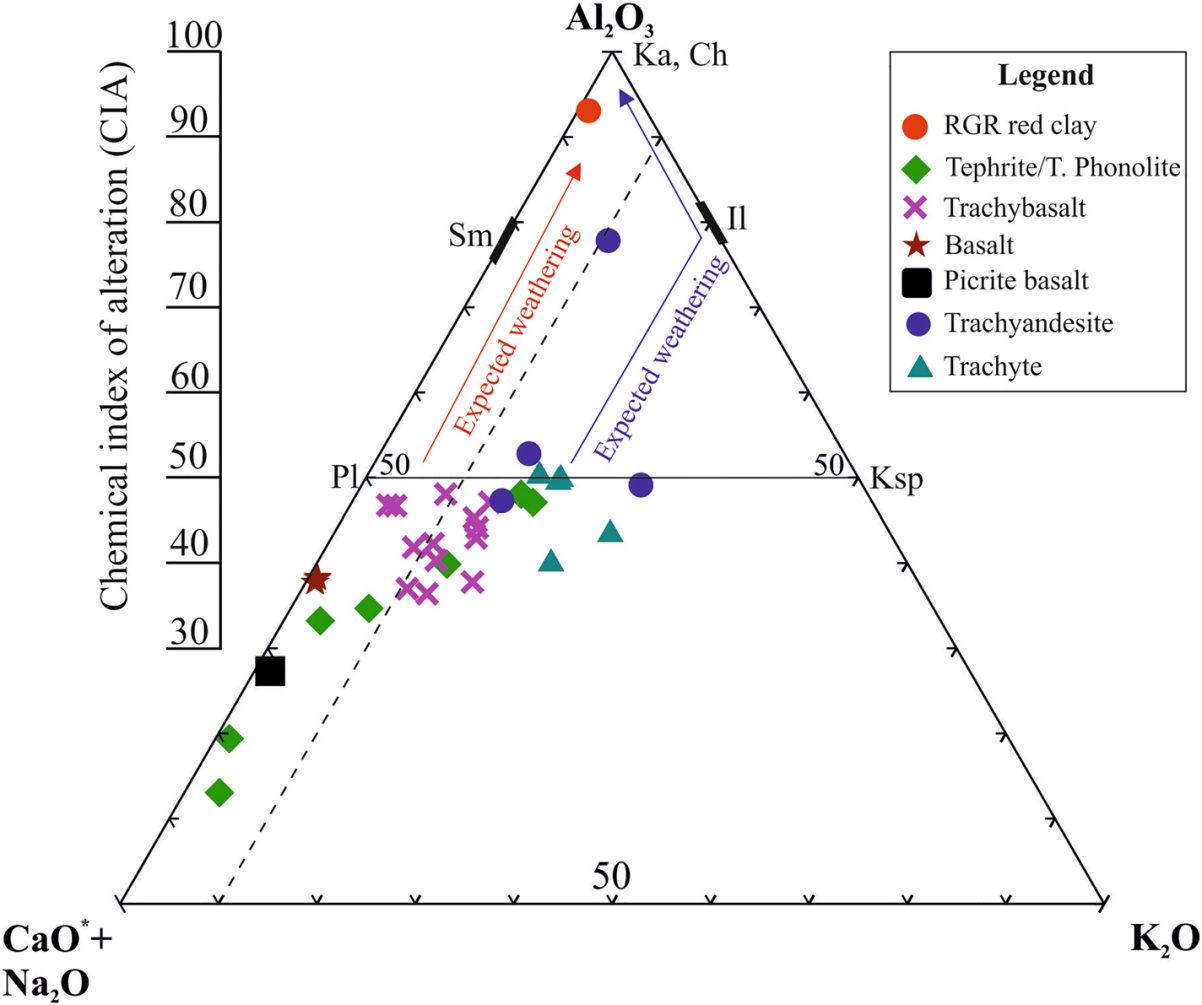
Ternary diagram showing the molar A-CN-K compositional space of weathered red clay sample along with relatively unaltered/fresh volcanic rocks from the WRGR. The major element data for DSDP Site 516 (basalts) and different alkaline volcanic rocks (e.g., tephrite, trachybasalt and trachyandesite) are from Hoyer et al.^10^ and Guerra et al.^43^. The major element data used in the A-CN-K calculations are presented in Table S3. The abbreviations are Pl, plagioclase; Ksp, k-feldspar; Sm, smectite; Il, illite; ka, kaolinite; Ch, chlorite.

The WRGR red clay sample data, plotted in the compositional space of the molar ternary plot Al_2_O_3_-CaO + Na_2_O + K_2_O (A-CN-K) along with the tholeiitic basalts of DSDP Site 516 (Late Cretaceous volcanism) and Eocene alkaline volcanic rocks recovered from the crest of the WRGR, is shown in Fig. 5. The different volcanic rocks of the WRGR, considered as parent/progenitor rocks for the red clay, fall on/or parallel to the A-CN axis (Fig. 5). The CIA of most of these alkaline rocks are < 50, and LOI < 5% indicating unaltered or weakly altered nature of these rocks (Table S3). However, some of these dredged alkaline rocks have higher CIA and LOI (e.g., trachyandesite recovered from RGR1-D05-001 dredged site) and have undergone moderate alteration (Fig. 5 and Table S3).

The red clay falls on the A-CN axis of plagioclase destruction (Fig. 5). The trachyte, some of the tephrite/phonolite and trachyandesite rocks follow the illite weathering trend, whereas picro-basalt and trachybasalt follow the kaolinite weathering trend and are most likely progenitor rocks for the red clay (Fig. 5).

### Rock magnetism

**Figure 6:**
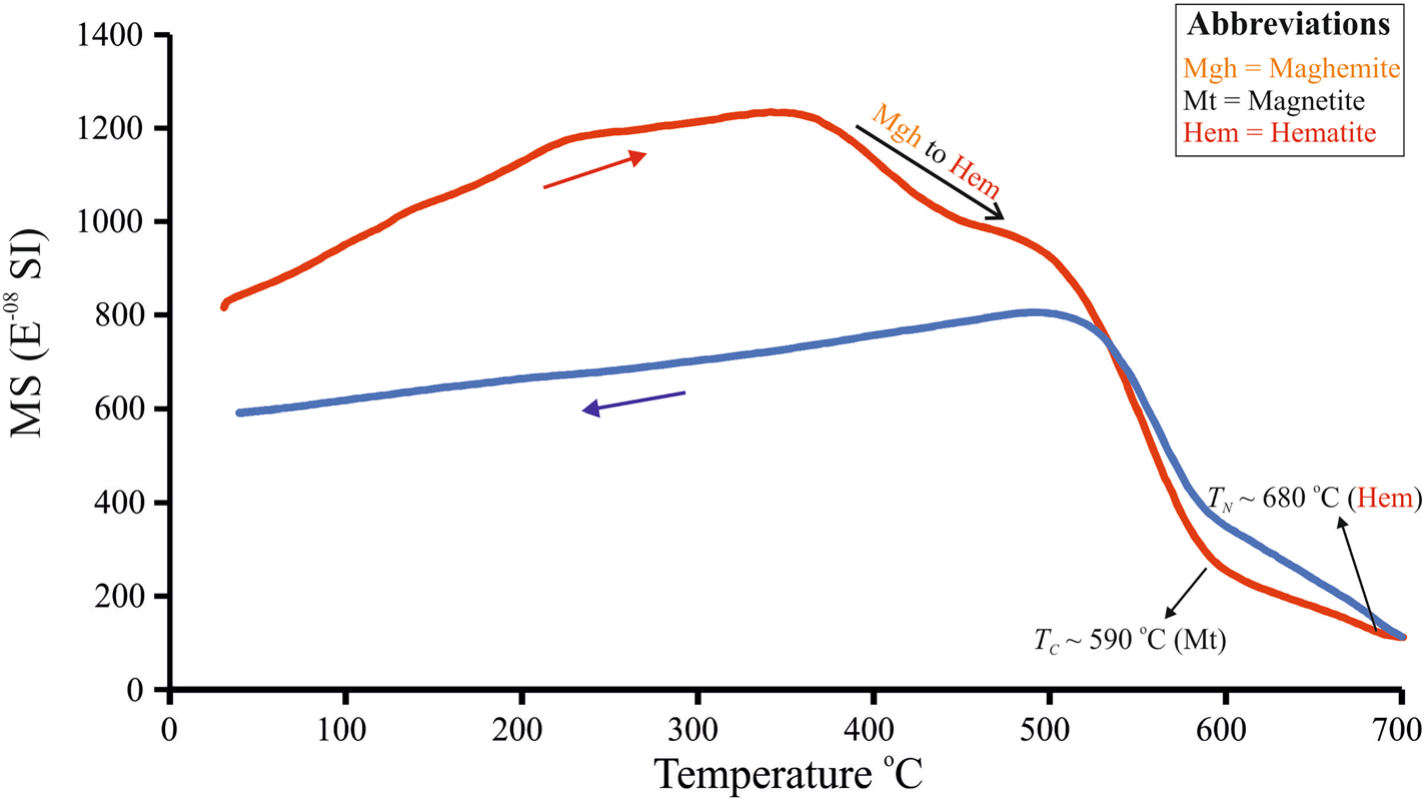
Temperature dependent magnetic susceptibility curve of the bulk red clay sample.

The χ-T analysis of the bulk red clay sample showed Curie (*T*_*C*_) and Néel (*T*_*N*_) temperatures at ~ 590 °C and ~ 680 °C indicating the presence of magnetite and hematite, respectively (Fig. 6). However, complete loss of magnetization was not seen up to ~ 700 °C. The decline in susceptibility from ~ 400 to 500 °C indicates a conversion of metastable maghemite to hematite (Fig. 6).

**Figure 7:**
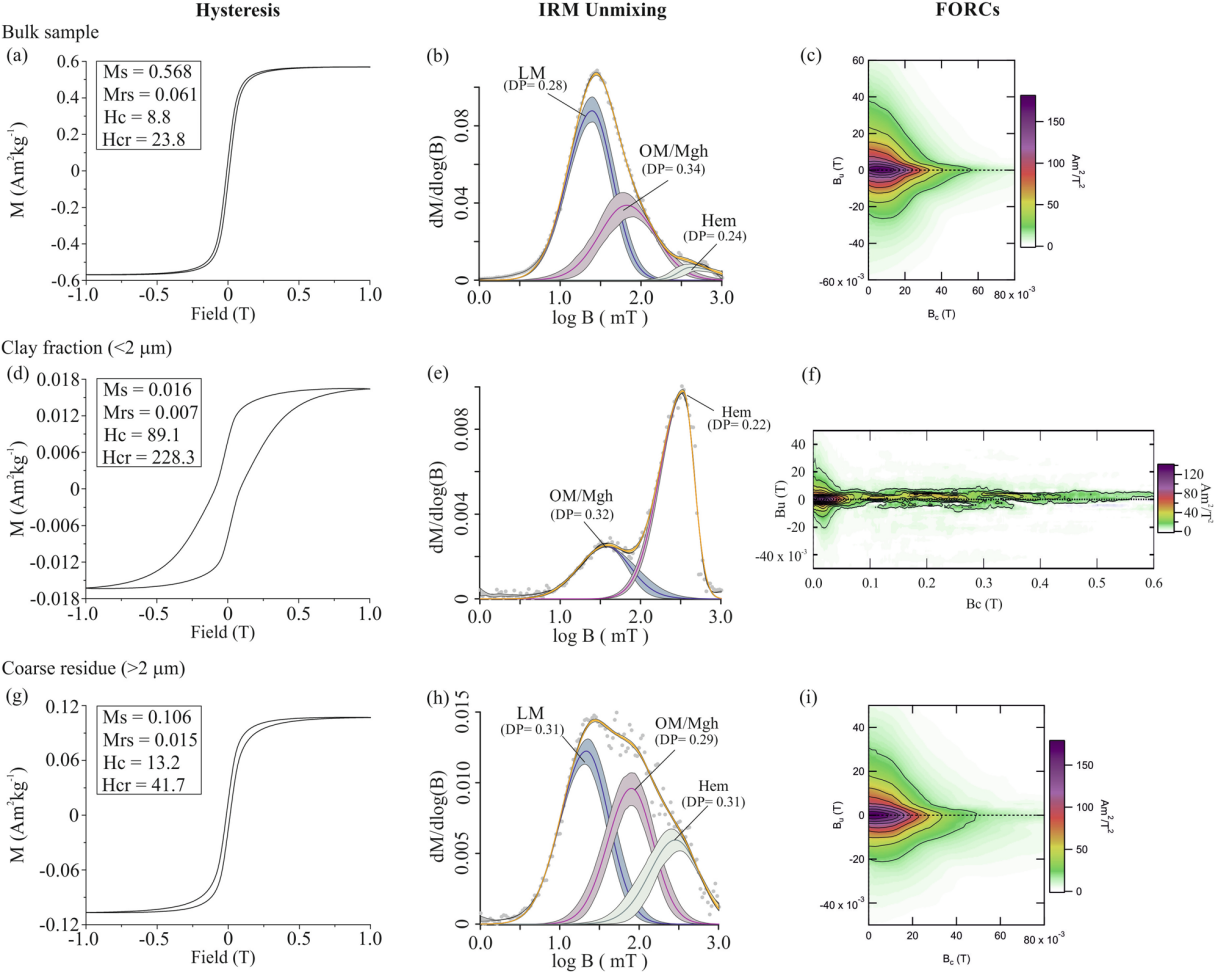
Hysteresis, IRM-Unmixing and FORC results on bulk red clay sample (**a**–**c**), separated clay fraction < 2 μm (**d**–**f**), and coarse residue fraction > 2 μm (**g**–**i**).

Hysteresis, IRM-unmixing, and FORC data of bulk, clay (< 2 μm), and coarse residue (> 2 μm) fractions are presented in Fig. 7. Bulk sample showed a narrow-constricted hysteresis loop closing well below 500 mT, typical of low-coercivity coarse ferrimagnetic minerals (Fig. 7a). The IRM-unmixing results on bulk sample showed three magnetic components (C1, C2 and C3). The mean coercivity (B_1/2_) and dispersion parameter (DP) values of the different components are provided in Table S4. Based on mean coercivity and dispersion values, C1, C2, and C3 components are identified as lithogenic magnetite, maghemite, and hematite, respectively (Fig. 7b). FORC result for the bulk sample showed a mixture of single domain (SD) and pseudo single domain (PSD)/vortex particles. The SD end member could be identified by the oval shape of contours along Bu = 0. The PSD end member was identified from the divergent contours in the upper half of the diagram and a smaller negative amplitude around the lower diagonal (Fig. 7c).

The clay fraction showed significantly different magnetic behavior than the bulk sediment. Hysteresis loop showed wasp-waisted behavior with no saturation up to an applied field of 1 T, indicating mixed low and high coercivity magnetic minerals (Fig. 7d). The IRM unmixing results showed only intermediate and high coercivity components assigned to maghemite and hematite, respectively (Fig. 7e). FORC result showed the mixed SD and PSD magnetic particles (Fig. 7f). The SD grains were identified by oval shape of contours along the Bu = 0 and a central ridge up to a higher coercivity end (~ 90 mT). The central ridge contribution to total magnetization was higher in the clay fraction. The high coercivity tail ranging up to 600 mT was from the hematite mineral contribution (Fig. 7f).

The hysteresis loop, IRM unmixing, and FORC result of coarse residue fraction (> 2 μm) showed similar behavior as of bulk sample (Fig. 7g–i). However, slight changes in mean coercivity and dispersion parameters of different magnetic components or in the hysteresis loop of the residual fraction compared to bulk sample are due to the extraction of magnetic minerals associated with clay fraction and/or due to the removal of finer magnetic particles during the chemical treatment.

## Discussion

The WRGR volcanic plateau records at least two magmatism episodes i.e., (1) late Cretaceous tholeiitic magmatism which occurred near the MAR, and (2) middle Eocene alkaline OIB type magmatism which led to formations of guyots and seamounts^3,5,8–10,20^. The discovery of the red and brown clay horizons (a few cm to > 10 cm thick) between successive alkaline lava flows (Fig. 2) indicates periods of hiatus between volcanic eruptions during the middle Eocene magmatic event. Similar clay-rich horizons are often reported as paleo-weathering surfaces, or paleosols, trapped between lava flows from numerous continental volcanic provinces, e.g., Deccan volcanic province^21–23^ and Columbia flood basalts^24^. These paleoweathering surfaces are often ascribed to sub-aerial weathering or pedogenic alterations of underlying volcanic rocks. Here, we evaluate whether the inter-lava flow horizons of kaolinite-hematite rich red and brown clays, from the WRGR, have also formed during sub-aerial exposure and weathering of the underlying alkaline lavas during pauses in volcanic eruptions, or were they deposited on top of lava flows, as detrital pelagic sediment derived from nearby continental landmasses (i.e., South America), during pauses in submarine volcanic eruption.

Over the past five decades, extensive studies on the clay mineral constituents of deep-sea surface/abyssal sediments of the South Atlantic have been carried out in order to understand the terrigenous sediment provenance, their mode of transportations (river, aeolian and glacial-marine), re-suspension and re-distribution by ocean circulations and climatic controls [e.g.,^25–27^]. The clay mineral distribution in the Atlantic shows a strong latitudinal zonation^25,26^, with a high concentration of kaolinite between 25° N and 25° S. Kaolinite sources in the Atlantic Ocean have been linked to the pedogenic/lateritic-bauxite alteration of the surrounding tropical landmasses in Brazil and western Africa that were transported by rivers and wind^25–27^. The high concentration of kaolinite in the Vema Channel and RGR region has been previously reported by Chamley^28^ and Jones^29^. The advective transport of kaolinite in the Vema Channel and RGR region was proposed either as a result of the southward flowing North Atlantic Deep Water (NADW)^28,30^ or through the isopycnal transport from the kaolinite rich sediments of the São Paulo Plateau^29^. Doce River was suggested as a prime contributor of the kaolinite rich sediments to the São Paulo Plateau^29^. Gingele et al.^31^ studied surface sediments and core samples collected at intermediate depths (~ 3000–4300 m) across the São Paulo Plateau to MAR covering the Vema Channel and western and eastern RGR. They reported abundant kaolinite in the surface and core samples of the Vema Channel, and of western and eastern flank of the RGR. Gingele et al.^31^ suggested injection of kaolinite-rich suspensions into intermediate depths of the NADW off the mouth of the Doce River and then advection with NADW to RGR rather than re-suspension of kaolinite rich sediments at São Paulo Plateau.

It is of utmost importance to note that the surface samples in these studies were collected at intermediate depths (≥ 3000 m), whereas the kaolinite-rich red clay observed and recovered in the present study are at ~ 650 m water depth and, therefore, could not have been transported as detrital clays through this deep-water mechanism (Fig. S7). Alternatively, Glasby^32^ suggested aeolian inputs as the major source of the fine lithogenous material for the surface pelagic red clays of the Pacific Ocean. The major potential source of modern-day dust in the southwestern Atlantic is Patagonia, with aeolian sediments derived from abundant loess deposits that are rich in titanomagnetite and illite^31^. Such aeolian dust is more prevalent during glacial periods when surface conditions are dryer, yet the desertification of central Patagonia only began during the middle Miocene (~ 12–14 Ma)^33^ and it is therefore unlikely to have contributed to the genesis of the red clay trapped between the middle Eocene alkaline volcanic rocks of the RGR.

Sub-aerial alteration of volcanic rocks and production of clay (mostly smectite) horizons at oceanic rises and plateaus have been previously reported from the North Atlantic Ocean (e.g., the Rockall Plateau) as well as from the South Atlantic (MAR and WR)^34–36^. These studies show an abundance of well-crystallized volcanogenic smectites at the basalt-sediment contact and a decrease in their abundance and crystallinity in the sedimentary column with increasing distance above the basalt. The locally active sub-aerial volcanism during the evolution of South Atlantic, and subsequent weathering and erosion may have significantly increased contribution of such volcanogenic clays in the sedimentary composition the South Atlantic basin^36^.

The sedimentary (clay and tephra-volcanic ash) records from the DSDP Sites 516 and 357 in Rio Grande Rise have shown extensive volcanic activity during the early middle Eocene (50–48 Ma), which diminished from the late Eocene to Oligocene (46–38 Ma)^15,20,37,38^. Bryan and Duncan^20^ dated alkaline volcanic ash deposit recovered from the DSDP Site 516 using the K–Ar method and reported an age of ~ 46.3 ± 0.7 Ma. They suggested alkaline volcanism at RGR as the most probable source of the sub-aerial volcanic ash deposition. Rohde et al.^9^ dated a phonotephrite dredged from a seamount in the WRGR using the K–Ar method and reported an age of ~ 46.0 ± 0.1 Ma. U–Pb dating of a trachyte recovered from the neighboring dredge (DR05) to the red clay has shown an age of ~ 43.9 ± 1.4 Ma (Fig. 1b)^12^. The chronology of the alkaline volcanic ash and volcanic rocks dredged from the WRGR seamounts supports the tectonic and sedimentary evolution model of the WRGR which indicates that a thermal anomaly created uplift and a bulge during the middle Eocene, giving elevation to the WRGR main platform, followed by intrusive and extrusive alkalic magmatism [e.g.,^5,11^]. Some of the volcanoes emerged as islands during this middle Eocene event, followed by extensive sub-aerial erosion and thermal subsidence and submergence during the late Eocene and early Oligocene (35–25 Ma)^11^.

The kaolinite is a common clay mineral found in the soils, lateritic regolith and paleosols that have developed in sub-aerial environments from extreme alteration/chemical weathering of parent rocks having dominant aluminosilicates mineral composition (e.g., feldspar), in a warm-humid climate with alternate wet and dry seasonality. Hydrolysis leads to the leaching of mobile alkali- and alkaline-earth elements (e.g., Na, Ca, Mg, K) from parent/progenitor rocks and residual immobile elements Al and Si lead to the formation of kaolinite. During the middle Eocene (50–40 Ma), the RGR falls into subtropical humid climatic zone of the mid-latitude (~ 40.6 to 38.5° S)^39^. The sub-aerially exposed alkaline volcanic rocks during this period are likely to have been exposed to greater degrees of chemical weathering and leaching of alkali- and alkaline-earth elements due to uplifted relief of the WRGR and warmer climatic conditions^40^, favoring the formation of kaolinite-rich red clay. In addition, the rock magnetic properties of the red clay showed the presence of primary magnetite and secondary maghemite and hematite minerals composition. The rock magnetic data of the WRGR volcanic rocks showed dominant primary titano-magnetite and magnetite compositions of different domain sizes (e.g., PSD and SD) (details in supplementary file). The weathering of volcanic rocks in a sub-aerial oxidizing environment alters coarser ferrimagnetic titano-(magnetite) particles into smaller fine superparamagnetic (SP)-SD ferrimagnetic particles and produces secondary iron oxides such as maghemite and hematite through oxidation and structural inversion^21,41^. The fine pigmentary hematite particles are often attributed to the red color of the soils and sediments, and can also form through chemical precipitation via hydrolysis during sub-aerial chemical weathering^41,42^. The magnetic analysis on clay separates indicates that the hematite is mainly associated with finer fractions in the WRGR red clay, and might have formed from the chemical precipitation during the chemical weathering of the WRGR volcanic rocks. The combined mineralogical, geochemical, and rock magnetic data of the red clay and AUV survey provides a strong evidence that WRGR red clay originated from the intense chemical weathering of the underlying volcanic rocks in a sub-aerial warm-humid environment during the middle Eocene.

## Conclusions

Mapping of vertical escarpments, formed in the wall of a major rift structure bisecting the WRGR, reveal horizontal lava flows and horizons of red clay trapped between successive flows at a water depth of ~ 650 m. This indicates either sub-aerial weathering of the lava flows, or pelagic terrigenous sedimentation, during pauses in the volcanic eruptions. Detailed mineralogical, geochemical, and rock magnetic analysis on the red clays, show kaolinite and hematite as dominant mineral composition. The fine hematite particles, giving red color to the clay, were formed from chemical precipitation via hydrolysis of volcanic rocks during humid chemical weathering and/or through oxidation of the primary magmatic titanomagnetite and magnetite. These characteristics are typical of red clays formed by extreme chemical weathering (CIA = 93) under warm and humid climatic conditions. The combined mineralogical, geochemical, and rock magnetic data of the red clay provides a strong evidence that WRGR red clay had originated from the intense chemical weathering of the volcanic rocks in a sub-aerial environment during the middle Eocene signifying the sub-aerial exposure of the RGR before thermal subsidence to its modern-day submerged depth.

## Material and methods

### Dredging and sample collection

Several sites were dredged on the WRGR, recovering a mixture of alkaline volcanic rocks, Fe–Mn crusts and nodules, and red clays^17^. The different sampling sites for the volcanic rocks and red clay are shown in Fig. 1b, and supplementary information on latitude, longitude, water depths, and lithologies recovered are provided in Table S1. Red clay sample RGR1_D09_006 was dredged from the western scarp of WRGR, at a water depth of ~ 650.5 m, and contained lithic fragments and foraminifera. Ten samples of volcanic rocks (n = 10) were also dredged and identified as trachyte, trachyandesite, trachybasalt and picro-basalt, based on petrography and whole-rock geochemistry^12,43^.

### X-ray diffraction (XRD)

The bulk and clay mineral compositions of the red clay sample were determined using the X-ray diffraction method. For bulk mineralogy, the sample was first air-dried and powdered (< 200 mesh) using a mortar and a pestle. The XRD scan was collected on a Bruker D8 Advance Diffractometer at Instituto de Energia e Ambiente, Universidade de São Paulo (USP), Brazil. The scans were run using a Cu anode with a voltage of 40 kV and intensity of 25 mA, collimator 2.5o, 0.02 mm Ni filter, automatic Air-scatter, at a scanning rate of 0.02° 2θ step size and 3.1 s acquisition time. X-ray pattern for the whole-rock sample was obtained under rotating conditions, between 2°and 100° 2θ, and using a 0.2° primary slit and 9.5 mm secondary slit.

For the clay mineralogy, clay fraction from the bulk sample was separated using the centrifuge settling method. The bulk sample was first disaggregated in distilled water and transferred to 1000 mL beakers. The sample was treated with 1N HCl, followed by H_2_O_2_ to dissolve carbonate and organic matter present. Sodium hexametaphosphate [(NaPO_3_)_6_] was added to the samples as a deflocculant. The sample was then sonicated and allowed to settle for ~ 1 h. The top 200 mL of suspended solution was collected through a micropipette, and the < 2 μm particle-size fraction was separated by centrifuge settling. The oriented mount of clays on glass slides was prepared and analyzed. The XRD scan on clay fraction was collected for air-dried, glycolated for at least 48 h, and calcined at 500 °C for 4 h in a muffle furnace treated sample for identification of clay minerals^44^. Clay fraction diffractograms were obtained for non-rotating samples, between 2° and 30° 2θ, and with a 0.5 mm primary slit and 1.0 mm secondary slit.

The processing of XRD diffractogram e.g., background determination, smoothening, and peak search was carried out using X’Pert High Score plus software. The peak identifications were carried out using Crystallography Open Database (COD) and American Mineralogist Crystal Structure database.

### Scanning electron microscopy and energy dispersive X-ray spectrometry (SEM–EDS)

The SEM imaging using backscatter electrons and energy dispersive X-ray chemical microanalysis were carried out on Pt-coated polished thin section of the red clay (highly friable). SEM images and X-ray microanalysis were obtained using a Quanta650 FEG electron microscope from FEI, equipped with an Energy Dispersion X-ray Spectrometer (EDS) Quantax 400 (technology SDD—Silicon Drift Detector) at Laboratório de Caracterização Tecnológica (LCT), USP, Brazil.

### Major element chemistry and loss on ignition (LOI)

The major oxide concentrations of the sample were analyzed using the fusion bead method of XRF on a Zetium model spectrometer (Malvern Panalytical) at LCT, USP, Brazil. Samples were first pulverized (< 200 mesh) in an agate mill to minimize metal contamination and then, ~ 1 g of fine powdered samples were fused with lithium tetraborate (Li_2_B_4_O_7_) to make a glass bead. The fusion bead method was applied to eliminate the effects of particle size and minimize matrix effects in analysis. The quality control of measurements was checked through the use of physical preparation replicates, analysis replicates and reference materials (international soil standards GBW 07401 and GBW 07404, and in-house standards Feldspar LCT-05 and Granite LCT-07). The accuracy of measurement with respect to the certified/working values of the standards was better than 5% for major oxides. The precision in terms of observed relative standard deviation (RSD) on repeated measurements is 1% for major oxides.

Approximately 1 g of fine powdered sample was heated at 1020 °C for 2 h using a muffle furnace to estimate LOI, and was presented as LOI (wt%) = 100 × (ash weight_1020°C_/sample weight).

The XRF and LOI were analyzed both on the bulk sample as well as on the carbonate free (1N HCl treated) sample for calculation of weathering intensity and carbonate content.

### Rock magnetism

The rock magnetic measurements i.e., temperature dependence of magnetic susceptibility (χ-T), hysteresis loop, first-order reversal curve (FORC), and isothermal remanent magnetization (IRM) were carried out on bulk red clay sample at Centro Oceanográfico de Registros Estratigráficos (CORE), Instituto Oceanográfico, USP, Brazil. The χ-T was measured up to a maximum temperature of 700 °C in an argon atmosphere using an AGICO furnace-equipped Kappabridge KLY-4 system. Hysteresis loop, FORC, and IRM acquisition were analyzed using a PMC Model 3900 Micromag vibrating sample magnetometer. A maximum of 1 T field was applied to trace the hysteresis loop. Saturation magnetization (Ms), saturation remanence (Mrs), and coercive force (Hc) were calculated from the slope (paramagnetic) corrected hysteresis cycle. The coercivity of remanence (Hcr) was calculated from the back-field remanence curves. The IRM acquisition data acquired at progressively higher DC fields (non-linear, initial field = 18 μT, slew rate = 0.1 s) up to a maximum field of 1 T in 150 steps were subjected to the IRM unmixing using the web application MAX UnMix^45^ for decomposition of various magnetic coercivity distributions. The 280 FORCs were measured for bulk red clay sample with an averaging time of 0.7 s and 0.1 s of slew rate between successive measurements. The saturation field of 1 T, and Hu and Hc max of 0.1 and 0.6 T, respectively, were applied for the FORC measurement. The FORC diagrams were processed using the Forcinel 3.0 software^46^ with smoothing factors of Sc0 = 7, Sc1 = 7, Sb1 = 7 and Sb0 = 3, horizontal and vertical lambda of 0.1 and output grid of 1.

Similar rock magnetic measurements were also analyzed for the clay (< 2 μm) and coarse residue (> 2 μm) fractions separated from the bulk sample. The same centrifuge settling method was adopted for the separation of clay fraction as was for the clay mineralogy.

Alkaline volcanic rocks (n = 10) recovered from the neighboring dredge sites were also analyzed for the magnetic mineral composition and domain characteristics. A maximum of 0.5 T field was applied for the hysteresis loop measurements of the volcanic rocks. The 125 FORCs were measured for each sample. The similar steps for the hysteresis and FORC processing were adopted as of the bulk red clay sample.

### Supplementary Information

Supplementary Information.

## Data Availability

All the data has been provided in the manuscript and supplementary file.
