# Supplementary material for: Red clays indicate sub-aerial exposure of the Rio Grande Rise during the Eocene volcanic episode
Source: Sci Rep. 2023 Nov 4;13:19092. doi: 10.1038/s41598-023-46273-y (PMC10625572; doi:10.1038/s41598-023-46273-y)
Supplement: Supplementary file 1 — Supplementary Information. [file 41598_2023_46273_MOESM1_ESM.pdf]

Supplementary Information

https://doi.org/10.1038/s41598-023-46273-y

Red clays indicate sub-aerial exposure of the Rio Grande Rise during the Eocene volcanic episode

Priyeshu Srivastava[1, 2, 3*], Bramley J. Murton[4], Lucy Gomes Sant'Anna[5], Fabio Florindo[3], Muhammad Bin Hassan[1], Julia Taciro Mandacaru Guerra[6], Valdecir de Assis Janasi[6], Luigi Jovane[1]

[1]Instituto Oceanográfico, Universidade de São Paulo, Praça do Oceanográfico, 191, São Paulo 05508-120, Brazil

[2]Indian Institute of Geomagnetism, Navi Mumbai 410218, India

[3]Istituto Nazionale di Geofisica e Vulcanologia, Via di Vigna Murata 605, Rome 00143, Italy

[4]National Oceanography Centre, European Way, Southampton SO14 3ZH, UK

[5]Instituto de Energia e Ambiente, Universidade de São Paulo, Av. Prof. Luciano Gualberto, 1289, São Paulo 05508-010, Brazil

[6]Instituto de Geociências, Universidade de São Paulo, Rua do Lago, 562, São Paulo 05508-080, Brazil

Email: priyeshu.s@iigm.res.in

1. Magnetic mineral composition of the WRGR alkaline volcanic rocks

The χ-T, hysteresis, and FORC results on 10 alkaline volcanic rocks including trachytes, trachyandesite, picro-basalt, and trachybasalt are presented in Figures S4-S6. The χ-T results showed titanomagnetite and magnetite mineralogy for different alkaline volcanic rocks with Curie temperatures ranging between ~540 and 590 °C (Fig. S4). The sample RGR1_D05_002 (trachyandesite) showed a Curie temperature of ~630 °C, which might represent partly oxidized magnetite (/maghemite) produced during alterations (Fig. S4). Several alkaline rocks also showed a hump-like signature between ~200 and 400 °C, which might indicate titanomagnetite/titanomaghemite minerals[47] or aggregates of the fine magnetic particles superparamagnetic (SP)-SD produced during alteration[48]. The RGR1_D05_001 (trachyandesite) showed unique and non-reversible curves with cooling curves having higher susceptibility indicating neo-formation of strongly magnetic minerals during the heating. This sample also has the highest CIA value = 78, compared to the other volcanic rocks, and shows maximum alteration (Table S3).

The hysteresis data showed narrow constricted closed loops for most of the volcanic rock samples acquiring saturation well below 500 mT indicating dominant coarse-grained ferrimagnetic mineral composition (Fig. S5). The RGR1_D05_001 (trachyandesite) sample showed relatively higher coercive force compared to the other volcanic rocks, and Mrs/Ms = 0.58 and Hcr/Hc = 1.19 values indicate single domain particle. Several trachyte rocks showed multi-domain behavior with the symmetrical upper and lower half of the FORC diagrams, low coercivity (Hc = <20 mT at the peak of the distribution), and vertical axis spread (Hu <40 mT) diverging away from the Hu = 0 axis (Fig. S6). The picro-basalt and trachybasalt (RGR1_D08_004 and RGR1_D13_002) showed asymmetrical upper and lower half of the FORC diagrams with low vertical spread (Hu <20 mT), typical of the PSD grains (Fig. S6).

The RGR1_D05_001 (trachyandesite) showed most unique FORC diagram with very strong negative interaction up to 80 mT on the Hc axis, while the Hu axis ranges from a few mT to 15 mT (Fig. S6). This sample showed SD magnetic particle behavior.

2. Supplementary Tables

Table S1. Location and water depth of red clay and volcanic rocks dredged during the R/V Alpha Crucis expedition in 2018. The rock type information is based on the detailed petrology and trace element analysis provided in Guerra et al.[43].

Sample ID	Latitude	Longitude	Depth (m)	Rock type
RGR1_D05_001	30.7964 °S	35.9799 °W	683	Trachyandesite
RGR1_D05_002	30.7964 °S	35.9799 °W	683	Trachyandesite
RGR1_D08_002	30.8464 °S	35.9923 °W	730	Trachyte
RGR1_D08_003	30.8464 °S	35.9923 °W	730	Trachyte
RGR1_D08_004	30.8464 °S	35.9923 °W	730	Picro-basalt
RGR1_D09_006	30.7939 °S	36.0233 °W	650.5	Red Clay
RGR1_D13_002	30.9343 °S	35.9734 °W	711	Trachybasalt
RGR1_D13_004	30.9343 °S	35.9734 °W	711	Trachyte
RGR1_D15_001	30.6190 °S	35.7768 °W	655	Trachyandesite
RGR1_D16_001	30.6375 °S	35.7390 °W	641	Trachyte
RGR1_D16_003	30.6375 °S	35.7390 °W	641	Trachyte

Table S2. The XRD results (d spacing) of bulk red clay sample.

**d-spacing (Å)**	**Minerals**
7.15, 4.47, 4.36; 4.18, 3.57, 2.33, 1.66, 1.45, 1.285, 1.237	Kaolinite
10.0	Illite
3.26, 2.82, 1.99, 1.41, 1.26	Halite
3.85, 3.03, 2.49, 2.09, 1.87, 1.42	Calcite
2.70, 2.52, 2.20, 1.84, 1.69, 1.60, 1.49	Hematite
2.57, 2.43, 2.24, 2.17, 1.91, 1.79, 1.55, 1.31	Goethite

Table S3. Major element chemistry and loss on Ignition data (LOI) data for different alkaline volcanic rocks and basalts (DSDP 516) used for the calculation of chemical weathering index (CIA). The sample details and data are after Hoyer et al.[10] and Guerra et al.[43].

Sample ID	Type of Rock	Al2O3	MgO	CaO	Na2O	K2O	P2O5	LOI	CIA	References
MSM82-24-DR-1	Tephrite	7.12	4.09	25.02	0.953	1.83	0.550	26.01	13	Hoyer et al. (2022)
MSM82-24-DR-1-A	Tephrite	10.61	4.52	21.60	3.65	0.729	0.752	13.96	19	Hoyer et al. (2022)
MSM82-24-DR-4	Tephrite	11.48	5.65	7.20	1.03	3.549	0.534	13.09	40	Hoyer et al. (2022)
MSM82-27-DR-2	Tephrite	13.44	10.78	11.42	2.44	2.866	1.05	3.72	35	Hoyer et al. (2022)
MSM82-27-DR-1	Tephrite	13.36	10.17	12.39	3.21	1.402	1.01	5.35	33	Hoyer et al. (2022)
MSM82-55-DR-1	tephri phonolite	17.70	2.27	4.96	4.04	5.717	1.11	4.79	48	Hoyer et al. (2022)
MSM82-55-DR-2	tephri phonolite	16.64	2.90	4.91	3.50	6.039	1.03	4.81	47	Hoyer et al. (2022)
MSM82-34-DR-2	Trachybasalt	16.47	6.65	8.00	3.06	1.525	0.991	6.31	47	Hoyer et al. (2022)
MSM82-34-DR-3-B	Trachybasalt	18.20	4.61	7.14	3.58	3.188	1.08	4.55	48	Hoyer et al. (2022)
MSM82-34-DR-3-C	Trachybasalt	15.33	5.29	8.01	3.28	3.027	0.787	2.43	42	Hoyer et al. (2022)
MSM82-55-DR-5	Trachybasalt	15.42	3.75	7.87	3.56	3.612	1.24	7.05	42	Hoyer et al. (2022)
MSM82-55-DR-6-B	Trachybasalt	16.96	2.36	6.42	4.31	5.377	0.840	5.70	43	Hoyer et al. (2022)
MSM82-55-DR-9	Trachybasalt	16.35	3.01	7.25	3.53	4.891	1.46	6.42	44	Hoyer et al. (2022)
MSM82-55-DR-16	Trachybasalt	17.29	2.37	6.38	4.45	4.735	1.28	5.47	45	Hoyer et al. (2022)
MSM82-57-DR-1	Trachybasalt	17.54	3.03	5.51	3.92	4.849	0.813	2.29	47	Hoyer et al. (2022)
MSM82-58-DR-1	Trachybasalt	11.25	10.76	7.57	2.36	3.018	0.706	2.96	37	Hoyer et al. (2022)
MSM82-58-DR-2	Trachybasalt	11.51	10.74	7.31	2.95	3.807	0.877	3.56	36	Hoyer et al. (2022)
MSM82-58-DR-3	Trachybasalt	11.94	10.29	6.42	2.82	3.281	0.894	3.69	40	Hoyer et al. (2022)
MSM82-58-DR-4	Trachybasalt	11.54	9.14	6.91	2.12	4.794	0.905	2.01	38	Hoyer et al. (2022)
RGR1-D13-002	Trachybasalt	17.73	5.2	7.44	4.33	1.35	0.77	4.54	47	Guerra et al. (2023)
128-1-23-24	Basalt	13.60	5.32	10.11	2.73	0.3495	0.32	3.28	38	Hoyer et al. (2022)
128-1-58-59	Basalt	13.45	5.35	9.82	2.92	0.3203	0.31	3.87	38	Hoyer et al. (2022)
128-1-124-125	Basalt	13.53	5.29	10.01	2.70	0.3146	0.49	4.00	38	Hoyer et al. (2022)
128-2-26-27	Basalt	13.40	5.42	9.87	2.79	0.3337	0.31	4.04	38	Hoyer et al. (2022)
128-2-37-38	Basalt	13.67	5.31	9.89	2.79	0.3383	0.31	3.91	38	Hoyer et al. (2022)
RGR1-D08-004A	Picro-basalt	11.01	8.96	14.29	2.23	0.59	0.41	2.90	27	Guerra et al. (2023)

MSM82-55-DR-14	Trachyandesite	19.23	1.13	2.98	4.91	5.089	0.751	3.07	53	Hoyer et al. (2022)
RGR1-D05-001	Trachyandesite	19.67	5.5	1.82	1.42	2.49	1.14	9.88	78	Guerra et al. (2023)
RGR1-D05-002	Trachyandesite	18.02	2.42	4.4	4.6	5.33	0.52	3.00	47	Guerra et al. (2023)
RGR1-D15-001	Trachyandesite	16.08	4.1	4.02	1.72	8.57	1.15	4.13	49	Guerra et al. (2023)
RGR1-D08-002	Trachyte	19.15	1.5	2.73	4.91	6.19	0.3	2.53	50	Guerra et al. (2023)
RGR1-D08-003	Trachyte	19.35	0.68	1.69	5.61	7.17	0.11	1.99	49	Guerra et al. (2023)
RGR1-D13-004A	Trachyte	18.29	0.59	1.39	5.46	6.76	0.15	1.02	50	Guerra et al. (2023)
RGR1-D16-001	Trachyte	16.6	0.59	6.25	2.68	9.17	0.29	6.32	40	Guerra et al. (2023)
RGR1-D16-003	Trachyte	17.25	0.76	4.44	2.45	10.34	0.32	4.73	43	Guerra et al. (2023)

Table S4. Results of the model fitting of the IRM unmixing using the Max Unmix method[45]. Bh = Mean coercivity, DP = Dispersion Parameter, P = Proportion factor, S = Skewness. The model results are in log units.

**Sample**	**Component 1 (C1)**				**Component 2 (C2)**				**Component 3 (C3)**			
	Bh	DP	P	S	Bh	DP	P	S	Bh	DP	P	S
Bulk Red Clay	1.35	0.28	0.82	0.91	1.84	0.35	0.36	1.02	2.75	0.24	0.06	1.47
Clay Separates	–	–	–	–	1.54	0.32	0.26	0.95	2.41	0.22	1.01	0.68
Coarse Residue	1.30	0.31	0.85	0.93	1.88	0.29	0.67	0.95	2.43	0.31	0.42	0.95

3. Supplementary Figures

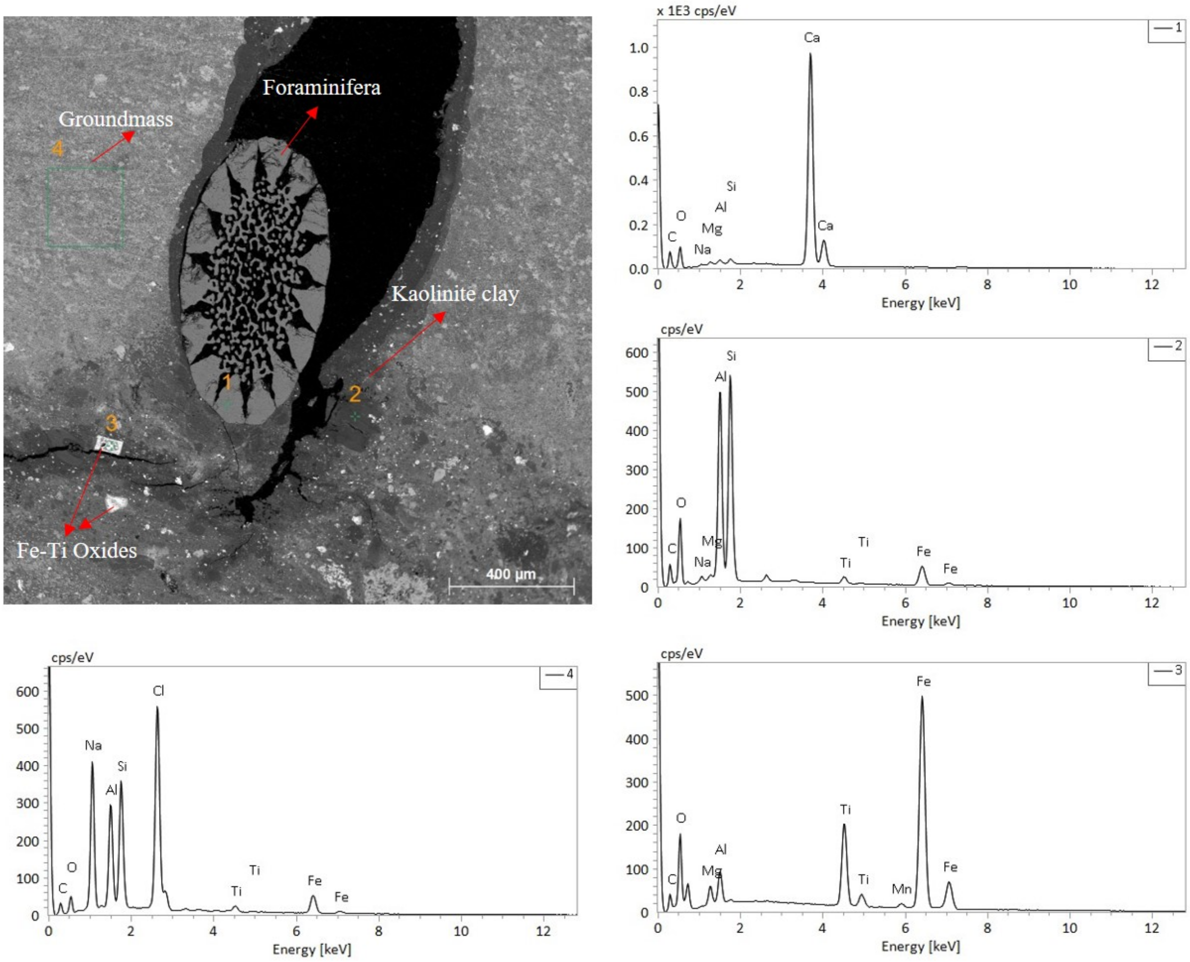

Figure S1. SEM image and EDX point analysis of the groundmass, lithic fragments and foraminifera assemble in the red clay sample. The foraminifera embedded in clay indicates its post-clay formational deposition.

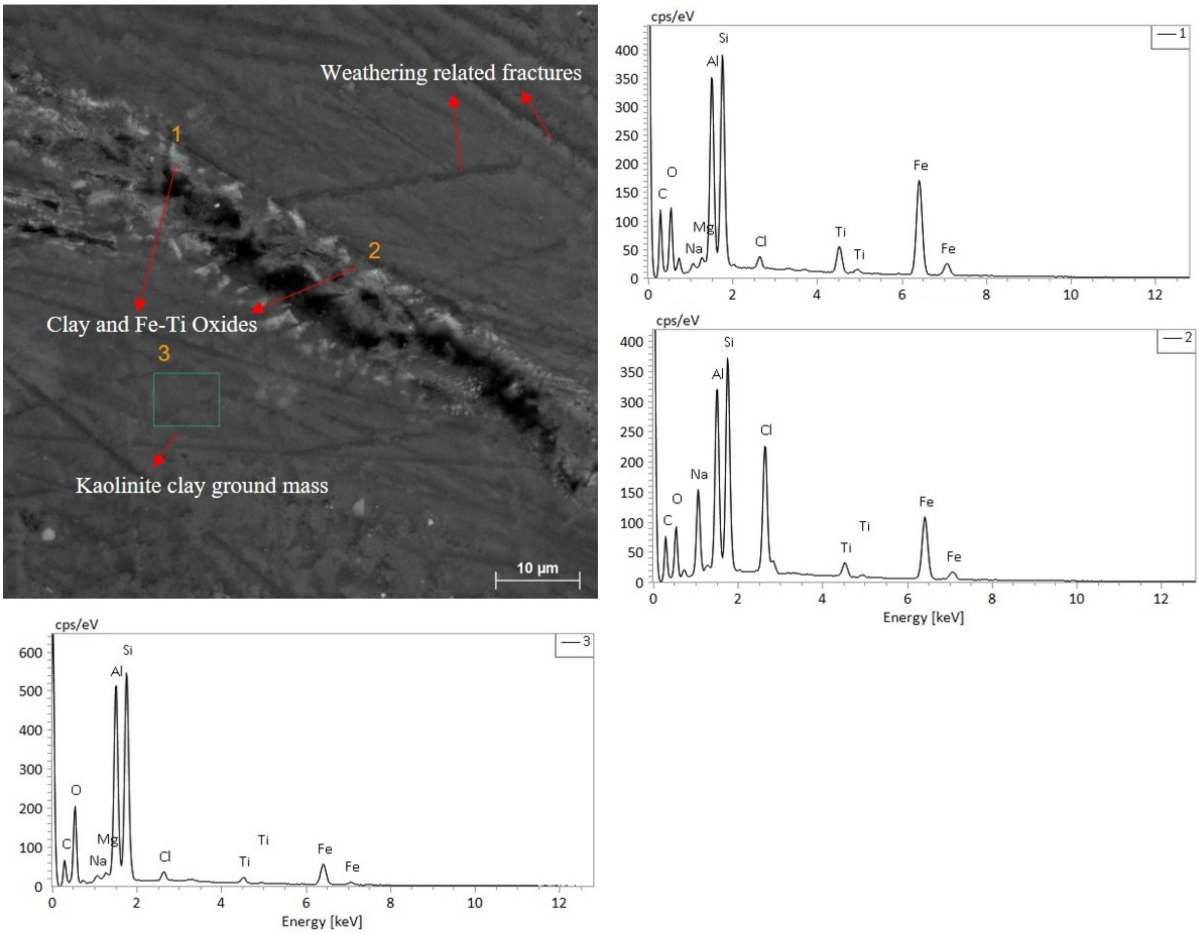

Figure S2. SEM image and EDX point analysis of the groundmass and lithic fragments along a weathering related fracture in the red clay sample.

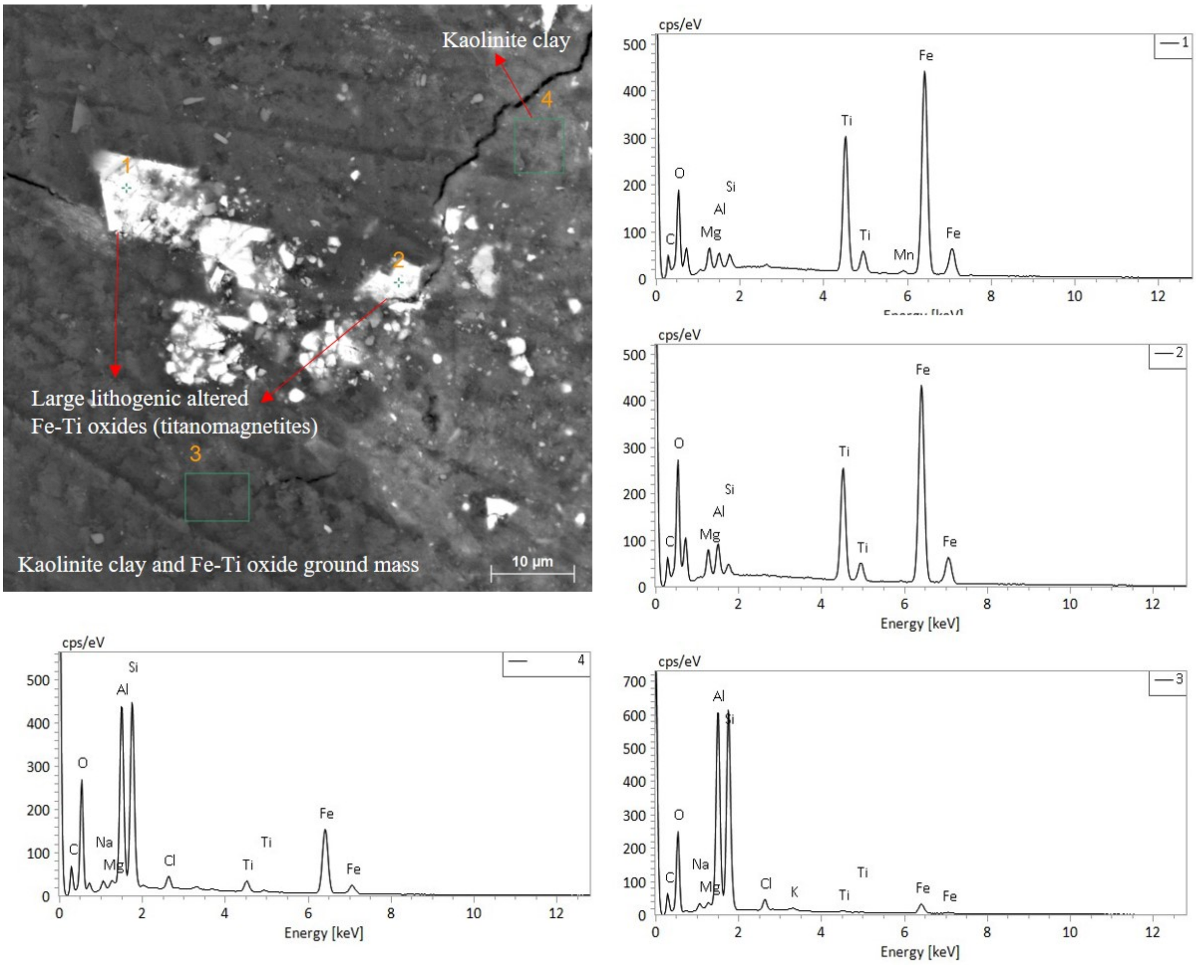

Figure S3. SEM image and EDX point analysis of the groundmass and coarse lithic fragments in the red clay sample. The coarse lithic fragments is composed of relatively weathering resistant minerals e.g., Fe-Ti oxides (titanomagnetites).

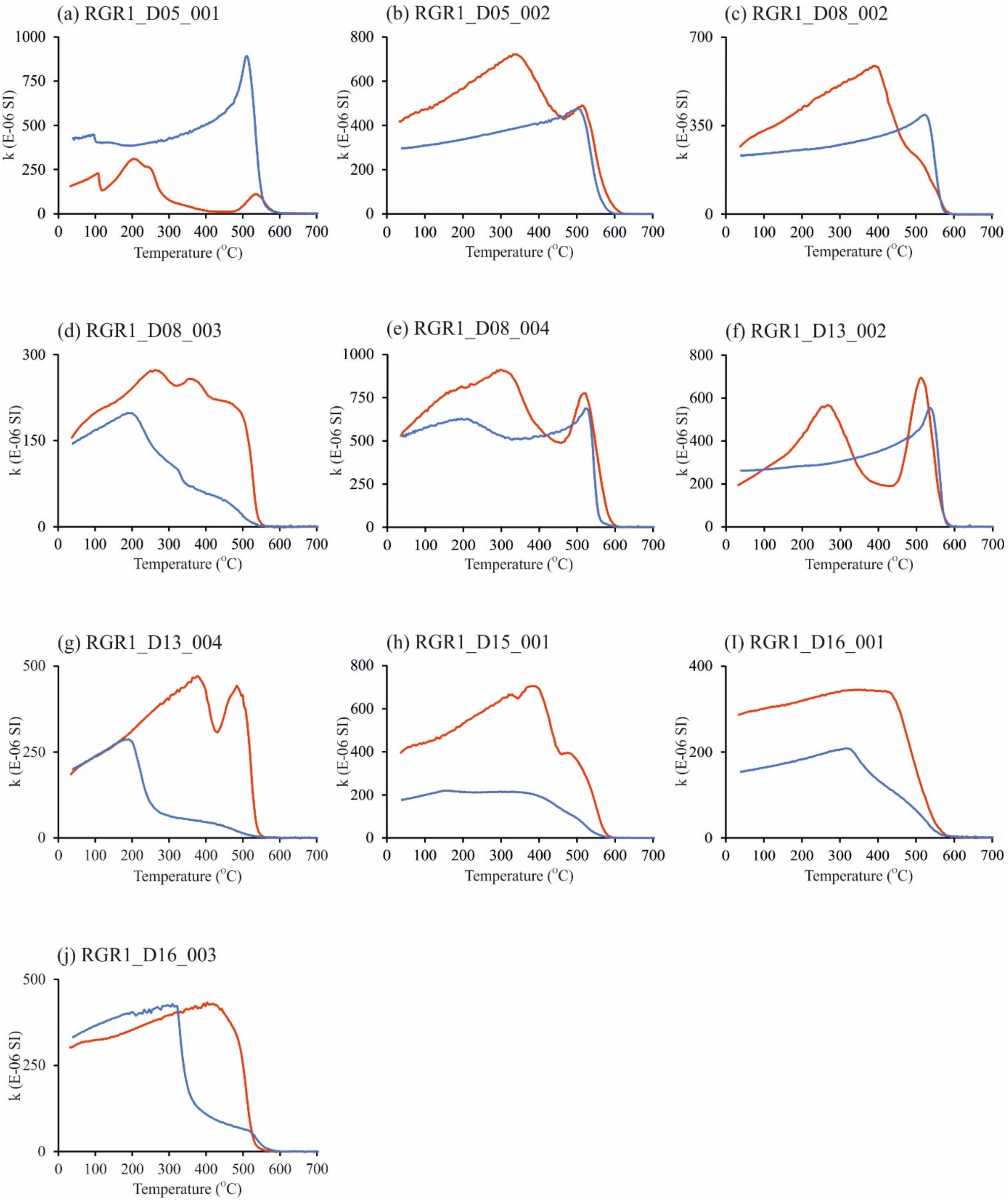

Figure S4. The χ-T results of the different alkaline volcanic rocks dredged from the WRGR. For the sample description see Table S1.

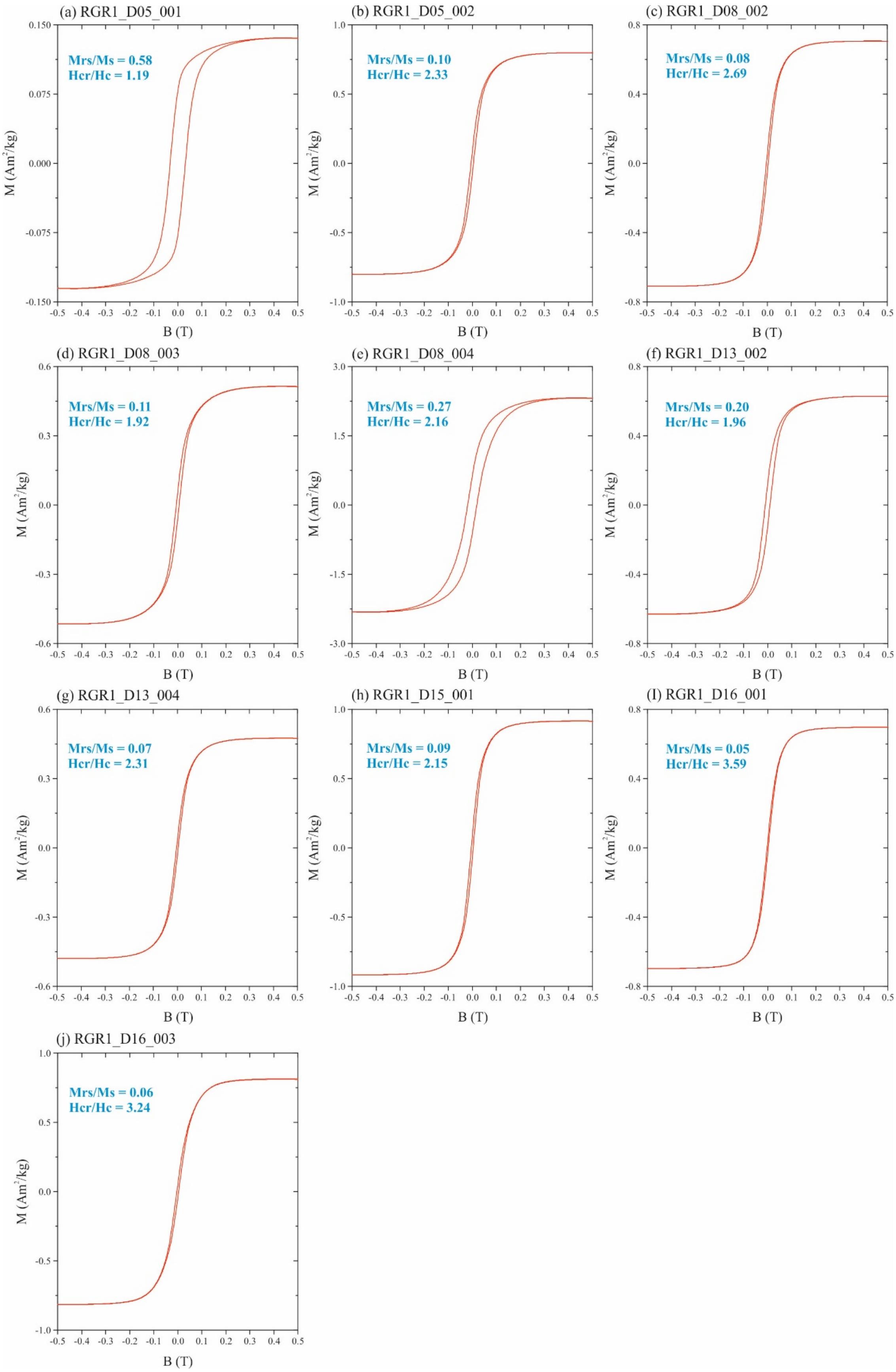

Figure S5. Hysteresis results of the alkaline volcanic rocks. The Mrs/Ms and Hcr/Hc values are also provided. For the sample description see Table S1.

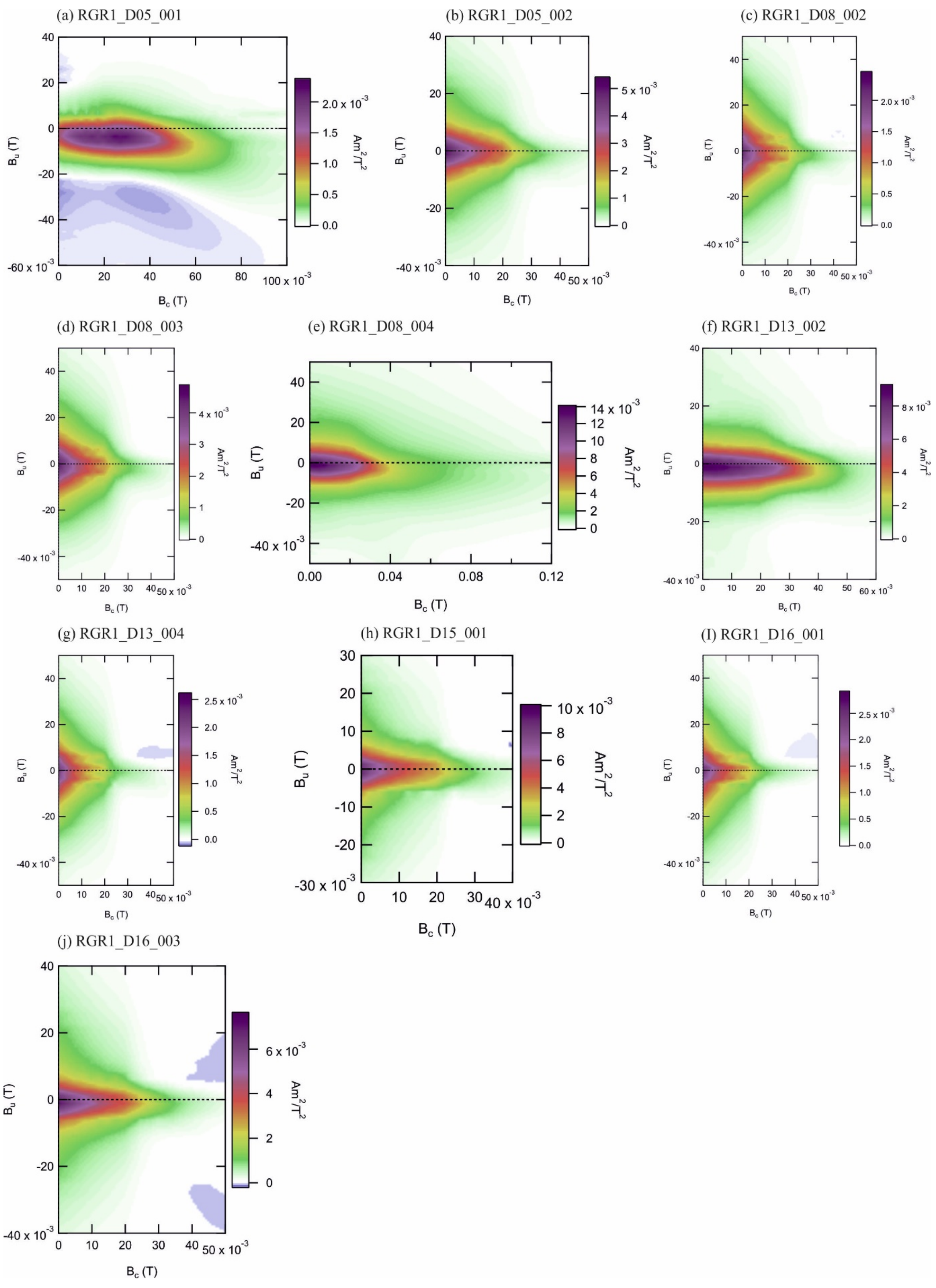

Figure S6. FORC results on different alkaline volcanic rocks recovered from the WRGR. For the sample description see Table S1.

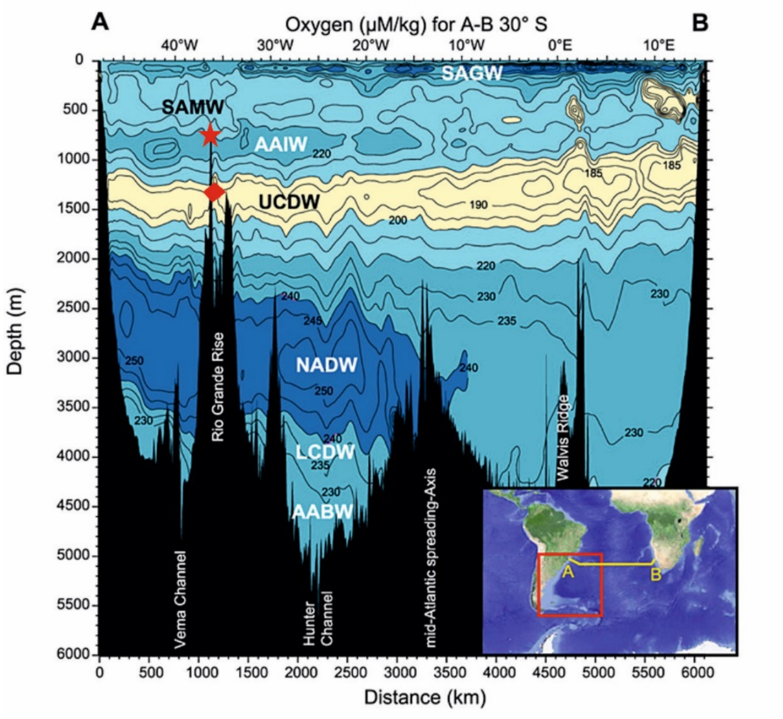

Figure S7. Vertical section of dissolved oxygen content (µM/kg) plotted as a function of depth, with vertical exaggeration (depth: distance) of 1:1000 (modified from the WOCE Atlantic Ocean Atlas) adopted from Florindo et al.[49]. Abbreviations: SAGW, South Atlantic Gyre Water; SAMW, Southern Annular Mode Water (~500 m deep); AAIW, Antarctic Intermediate Water (~700–1100 m deep); UCDW, Upper Circumpolar Deep Water (~1100–1600 m deep); NADW, North Atlantic Deep Water (~1600–3600 m deep); LCDW, Lower Circumpolar Deep Water (~ 3600–4500 m deep); and AABW, Antarctic Bottom Water (>4500 m deep). The star represents the depth of red clay and rectangle represents the DSDP Site 526.

References

47. Muxworthy, A. R. et al. Interpreting high-temperature magnetic susceptibility data of natural systems. Front. Earth Sci. 11, 1171200 (2023).

48. Zhang, Q. et al. Is alteration of magnetite during rock weathering climate-dependent?. J. Geophys. Res.: Solid Earth 126, e2021JB022693. https:// doi. org/ 10. 1029/ 2021J B0226 93 (2021).

49. Florindo, F. et al. New magnetobiostratigraphic chronology and paleoceanographic changes across the Oligocene-Miocene boundary at DSDP site 516 (Rio Grande Rise, SW Atlantic). Paleoceanography 30, 659–681 (2015).
